# Type 1 Brugada Pattern Unmasked During the Recovery Period of an Exercise Stress Test

**DOI:** 10.5935/abc.20160071

**Published:** 2016-05

**Authors:** Daniel García-Fuertes, Elena Villanueva-Fernández, Manuel Crespín-Crespín, Alberto Puchol, Marta Pachón, Miguel Angel Arias

**Affiliations:** 1 Department of Cardiology - Hospital Santa Bárbara, Puertollano, Ciudad Real; 2 Cardiac Arrhythmia and Electrophysiology Unit - Department of Cardiology - Hospital Virgen de la Salud, Toledo - España

**Keywords:** Hypertension, Cardiomyopathies, Defibrillators, Implantable, Kidney Neoplasms

## Introduction

Brugada syndrome (BrS) is an autosomal dominant channelopathy considered to be responsible for 4% to 12% of all sudden cardiac deaths and up to 20% of sudden cardiac deaths that occur in normal hearts.^1^ It is characterized by specific electrocardiographic findings in the right precordial leads. Although three patterns have been described, BrS is only diagnosed in patients with ST-segment elevation with type 1 morphology > 2 mm in at least 1 lead among the right precordial leads (V1, V2) occurring either spontaneously or after provocative drug test with intravenous administration of Class I antiarrhythmic drugs^2^.

Symptoms often occur during rest or sleep, during febrile state, or in vagotonic conditions, as the recovery period of an exercise test could be considered. In fact, attenuation of ST-segment elevation at the peak of exercise stress test followed by its appearance during the recovery phase is considered not only supportive for the diagnosis of BrS,^2^ but also as a possible predictor of adverse outcomes.^3^ These data have been obtained from series of patients previously diagnosed because of a spontaneous or pharmacologically-induced type 1 pattern, but cases without previous evidence of this pattern where the diagnosis was unmasked by exercise testing are scarce.

## Case Report

**Figure 1:**
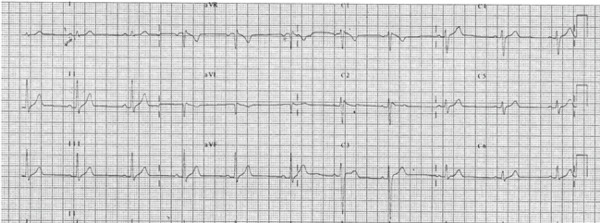
Twelve-lead ECG of the patient at baseline.

**Figure 2:**
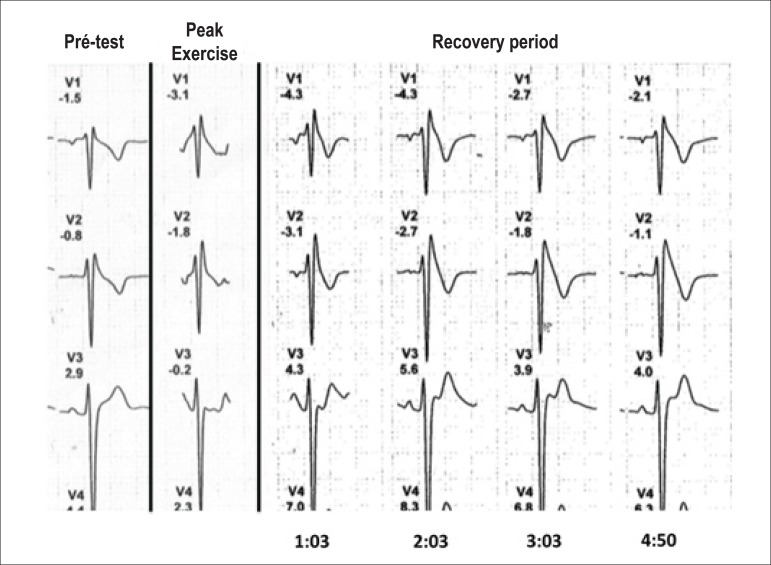
Electrocardiographic leads V1, V2 and V3 during exercise testing at baseline, at peak of exercise and during minutes 1, 2, 3 and 4 of the recovery period.

We report the case of a nineteen year-old male without personal or family history of cardiovascular disease or sudden cardiac death who was submitted to an exercise stress test because of atypical chest pain during exertion. He had never suffered syncope or palpitations. His resting electrocardiogram (ECG) showed a slightly elevated ST-segment in the right precordial leads without evidence of type 1 Brugada pattern, so it was classified as type III (Figure 1). An echocardiogram was performed and structural heart disease was ruled out. A treadmill exercise stress test using a standard Bruce protocol was performed due to persistent symptoms. The resting ECG before exertion was consistent with incomplete right bundle-branch block with an ST segment only elevated in V3 lead. No significant repolarization changes in the right precordial leads occurred during exertion. However, during the recovery phase, a J-point and coved ST segment elevation > 2 mm compatible with spontaneous type 1 Brugada pattern became evident in lead V2 (Figure 2). Additionally, intravenous pharmacological challenge with flecainide showed the appearance of a type 1 Brugada pattern. An electrophysiological study (EPS) was performed. At baseline, the type 1 Brugada pattern was not evident and conduction intervals were within the normal range (HV interval 46 ms). The EPS demonstrated the development of ventricular fibrillation during standard programmed electrical stimulation protocol (ventricular stimulation protocol was undertaken from the right ventricular apex at two basic drive cycle lengths of 600 ms and 400 ms, with up to three extrastimuli; in our patient ventricular fibrillation was induced during stimulation at 600 ms cycle length and 3 extrastimuli at 210, 200 and 200 ms). A subcutaneous implantable cardioverter defibrillator was implanted. All first-degree relatives had a normal resting ECG. Drug challenge test with flecainide and exercise stress test were performed to all of them, but no other type 1 Brugada pattern was induced.

## Discussion

To the best of our knowledge, only one case of Brugada type 1 pattern induction during the recovery phase of an exercise test in an asymptomatic patient without a family history of Brugada syndrome has been previously reported;^4^ other reported cases were diagnosed following the sudden death of a first-degree relative or in patients with previous syncopal episodes.^5^

Makimoto et al^3^ demonstrated that augmentation of ST-segment elevation during the recovery phase of a stress test occurred in 37% of BrS patients and that it was a predictor of poor prognosis, with arrhythmic events being more frequent in these patients, especially among those with a history of syncope and among asymptomatic patients. In addition, cases of ventricular arrhythmias during early recovery phase of the exercise test have also been reported.^5^ However, exercise testing is not considered a routine test for risk stratification in these patients. The role of stress test in the diagnosis and risk stratification of first-degree relatives without a previous spontaneous or induced type 1 pattern has not been assessed either.

Although it is known that autonomic function plays a main role, the exact mechanisms responsible for ST segment elevation after exertion in Brugada patients are not well established. Previous research has proven that the ST-segment elevation is mitigated by the administration of beta-adrenergic agonists and enhanced by parasympathetic agonists.^3^ Changes during the recovery phase of an exercise stress test appear to be similar to the exacerbation of the Brugada pattern seen with the administration of parasympathetic-stimulating agents.^6^ An increased parasympathetic basal activity or an increased susceptibility to the parasympathetic reactivation after exertion, with a simultaneous decrease of sympathetic stimulation is thought to influence this phenomenon.

Controversy exists about the prognostic value of ventricular arrhythmia inducibility during electrical programmed stimulation in asymptomatic patients with Brugada Syndrome. Expert consensus recommendations establish that implantable cardioverter defibrillator may be considered in patients with a diagnosis of Brugada Syndrome who develop ventricular fibrillation during programmed electrical stimulation.^2^ While some large registries have failed to demonstrate that inducibility predicts arrhythmic events,^7,8^ some other groups indicate that inducibility during EPS is an independent predictor for arrhythmic events, stressing also its negative predictive value.^9,10^
